# Massive Bilateral Haemorrhagic Prepatellar Bursitis: A Case Report

**DOI:** 10.7759/cureus.74351

**Published:** 2024-11-24

**Authors:** Adam Maguire, Christopher Lawrence, Paul Nicolai, Craig Rosenbloom

**Affiliations:** 1 Department of Trauma and Orthopaedics, West Suffolk NHS Foundation Trust, Bury St Edmunds, GBR; 2 Department of Trauma and Orthopaedics, Mid and South Essex NHS Foundation Trust, Chelmsford, GBR; 3 Department of Sports Medicine, Barts Health NHS Trust, London, GBR

**Keywords:** bilateral, bursitis, haemorrhagic, prepatellar, traumatic

## Abstract

Prepatellar bursitis is a common cause of anterior knee pain. We present an unusual case of massive bilateral traumatic haemorrhagic prepatellar bursitis managed with surgical excision and describe our operative findings. The patient presented with large bilateral knee swellings which had been present for six years following a fall onto both knees. The swellings interfered with his ability to wear trousers and lean up against ladders. Excision of the prepatellar swellings was performed sequentially. A thick-walled, well-demarcated cyst was excised from each knee, which was dissected from the surrounding tissues with relative ease. On the right, the contents of the cyst were entirely solid, consisting of organised haematoma (chocolate-coloured paste-like material), but on the left, a collection of thick, brown fluid was drained as well. More than two years following surgery, there has been no recurrence of the lesions. Chronic massive bilateral haemorrhagic prepatellar bursitis has not been previously reported, but in this case, it was successfully managed with surgical excision.

## Introduction

The prepatellar bursa is located subcutaneously and is a normal structure present in most people [1,2]. Its function is to facilitate movement of the skin over the anterior knee [3]. Prepatellar bursitis, also known as housemaid’s knee, is an occupational excessive fluid collection in the potential prepatellar bursal space and is a common cause of swelling and pain above the patella [4]. In the general population, bursitis is felt to be due to either acute trauma from a single blow or from chronic irritation [5]. The other causes of prepatellar bursitis include infection or low-grade inflammatory conditions, such as gout, syphilis, tuberculosis, or rheumatoid arthritis [2]. We present an unusual case of massive bilateral traumatic haemorrhagic prepatellar bursitis managed with surgical excision and describe our operative findings.

## Case presentation

A 60-year-old painter and decorator presented with a six-year history of substantial bilateral anterior knee swelling following a fall onto both knees from a minimal height. He was not taking any anti-coagulation medication. He complained of minimal pain and movement restriction, but the swellings interfered with his ability to wear trousers and lean up against ladders. The swellings had been aspirated without image guidance by his general practitioner numerous times but always re-accumulated, becoming larger with each subsequent recurrence.

**Figure 1:**
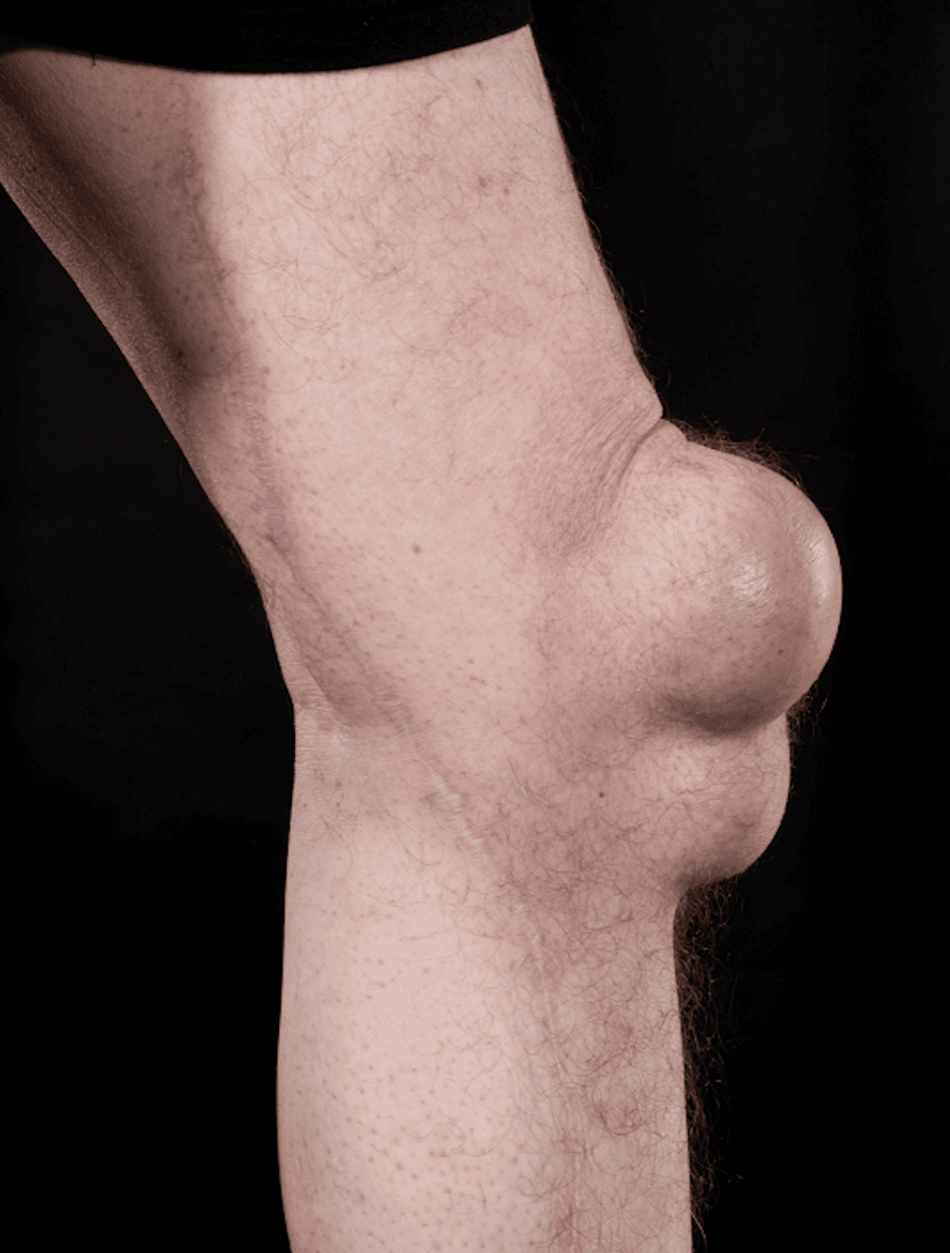
Pre-operative photograph showing the profile of the left knee prepatellar swelling.

Examination revealed firm, immobile masses on the anterior aspects of both knees approximately 15 cm by 10 cm, protruding 5 cm anteriorly with the right side being slightly larger than the left. Both appeared to be multi-lobular. There was no tenderness and both knees had a full range of motion. There were no inflammatory signs, and the skin was not compromised (Figure 1).

**Figure 2:**
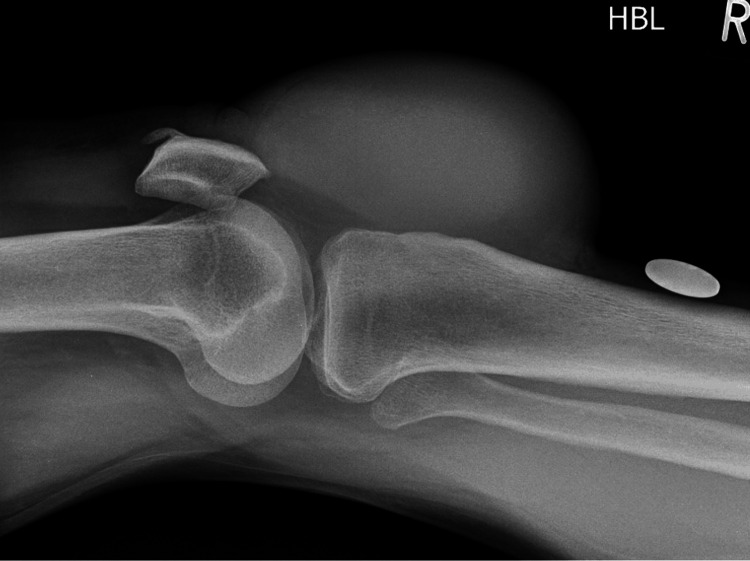
Right knee plain radiograph. HBL: horizontal beam lateral view; R: right

Knee radiographs showed no calcification (Figure 2). An ultrasound scan demonstrated bilateral cystic lesions incorporated with solid tissue. Blood testing did not reveal any platelet or coagulation disorders.

**Figure 3:**
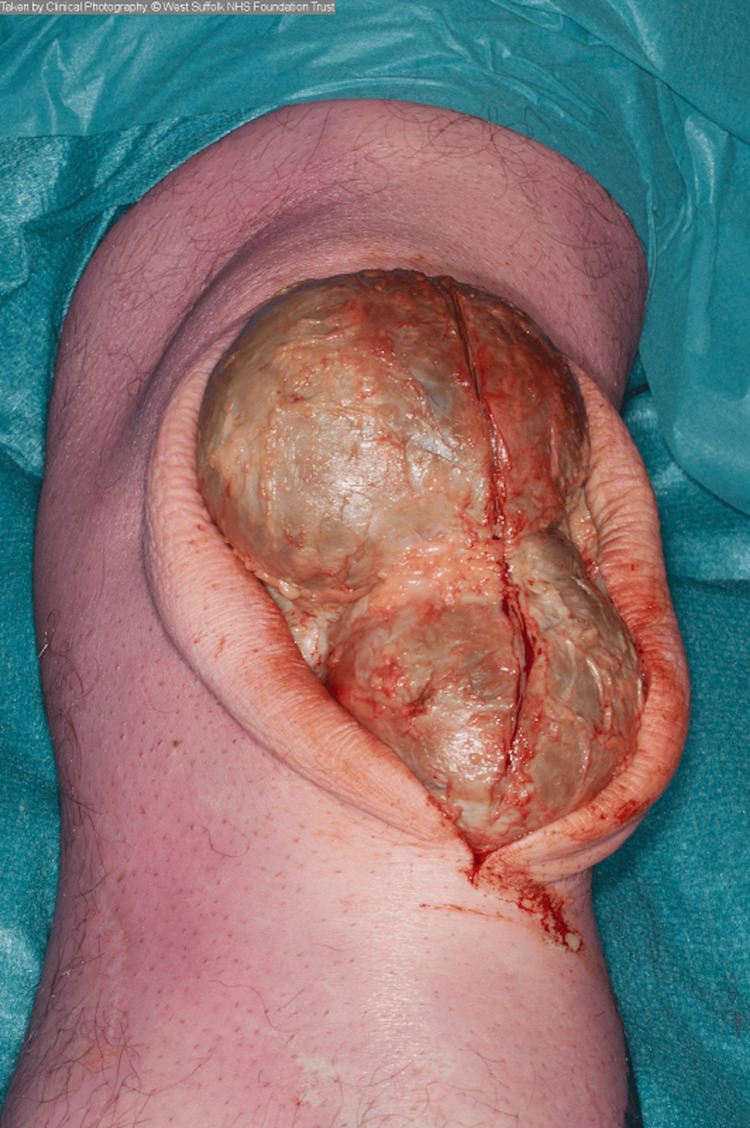
Intra-operative photograph showing the walled cyst following dissection from overlying soft tissues.

Excision of the prepatellar swellings was performed sequentially. A thick-walled, well-demarcated cyst was excised from each knee, which was dissected from the surrounding tissues with relative ease (Figure 3).

**Figure 4:**
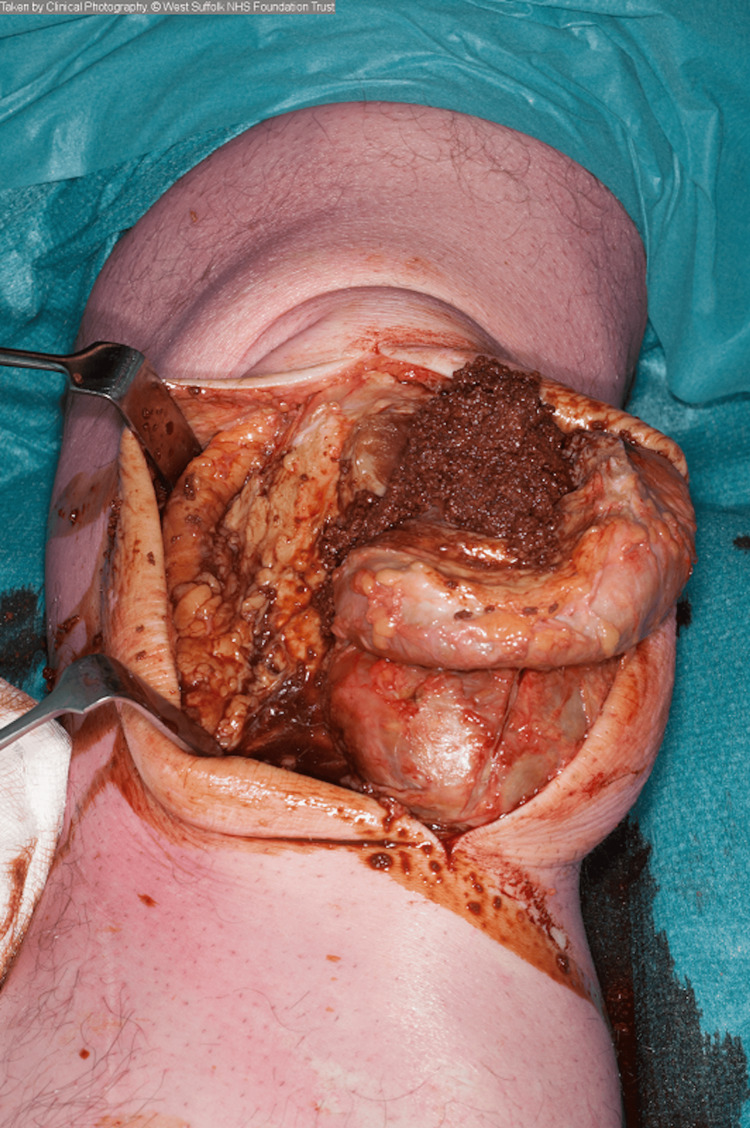
Intra-operative photograph showing chocolate-coloured paste-like substance and brown liquid found within cyst.

**Figure 5:**
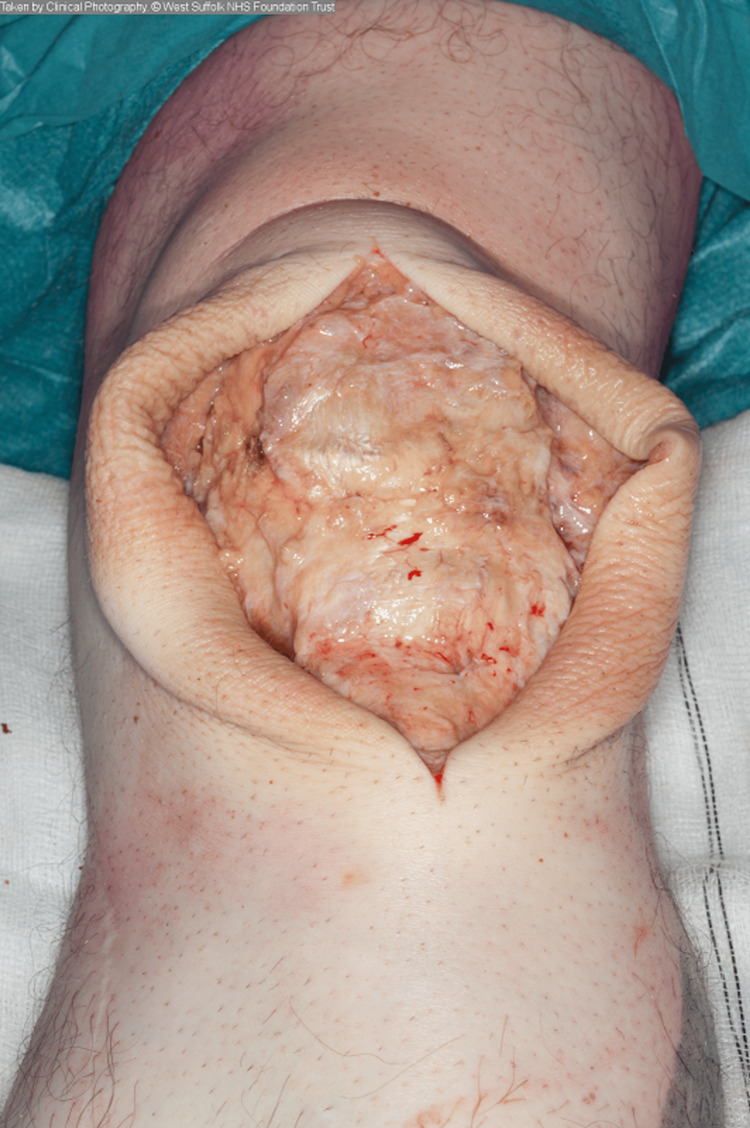
Intra-operative photograph following complete excision of cyst showing underlying patella and patella tendon.

On the right, the contents of the cyst were entirely solid, consisting of organised haematoma (chocolate-coloured paste-like material), but on the left, a collection of thick, brown fluid was drained as well (Figure 4). The cysts were completely excised, revealing the patella and patellar tendon below (Figure 5).

Microscopic examination revealed a sac-like structure comprised of hyalinised fibro-connective tissue filled with organised haematoma. There were no signs of malignancy or infection. The clinical, intra-operative and histopathologic findings were consistent with a diagnosis of chronic bilateral haemorrhagic prepatellar bursitis. To date, with over two years of follow-up, there has been no recurrence of the lesions.

## Discussion

Normal bursa is lined with large and delicate blood vessels which, upon direct trauma, can rupture causing a large acute prepatellar bursitis revealing gross blood upon aspiration [1,4]. However, it is thought that lesser degrees of repeated trauma could lead to an acute inflammatory response and excess bursa fluid production, and therefore a straw-coloured aspirate [6].

Prepatellar bursitis is a common cause of anterior knee pain. Differentials of this condition include patellar subluxation/dislocation, apophysitis, tendonitis, patellar tracking disorders, arthritis (osteoarthritis, rheumatoid arthritis, or septic arthritis), gout and pseudogout, cellulitis/other soft tissue infections, and rarely, neoplasms such as pigmented villonodular synovitis (PVNS) or local bone or soft tissue tumours. It is therefore important to examine the patient thoroughly, use appropriate imaging, and, if necessary, perform a biopsy for histopathologic analysis. Management of prepatellar bursitis is usually treatment of the acute symptoms with ice, compression, anti-inflammatory medication, immobilisation and antibiotic treatment for severe inflammatory or infected cases. Surgical excision of bursae is not commonly recommended or required, and there is no consensus on the optimal treatment of prepatellar bursitis [7].

In our case, the patient experienced direct trauma during a fall six years prior to the bursectomy; still, the contents of the bursa were haematoma. It was previously reported that the more long-standing the swelling of the bursa, the less likelihood that blood would be found at any acute recurrence [1]. Our patient’s bursae were aspirated many times, yet they reoccurred with the contents being organised haematoma. This could be explained by the larger molecularly-weighted breakdown products of blood having difficulty passing through the synovial membrane [1]. In addition, it is suspected that the occupational factor (i.e., repetitive bursae trauma or irritation through direct loading in a kneeling position) has led to chronic inflammation in the bursae wall, thereby increased difficulty in blood products being absorbed by the synovial membrane.

Chronic bilateral haemorrhagic prepatellar bursitis has not been reported in the medical literature. Cases of unilateral haemorrhagic prepatellar bursitis have been reported [4,8], and there have been cases of bilateral prepatellar bursitis reported in the past, but these have never shown a haemorrhagic component on the background of previous trauma and chronic occupational irritation [3,5,9]. These reported cases were also managed symptomatically with conservative measures.

## Conclusions

Chronic massive bilateral haemorrhagic prepatellar bursitis has not been previously reported, but the patient in the present case was successfully managed with open surgical excision following a number of attempts at aspiration. Haemorrhagic prepatellar bursitis should be considered a differential diagnosis of bilateral anterior knee swelling, particularly when there is a history of previous trauma or chronic occupational irritation. Surgical excision should be a consideration when the lesions are persistent and interfering with activities of daily living and occupation.
